# What is the impact of artificial intelligence-based chatbots on infodemic management?

**DOI:** 10.3389/fpubh.2024.1310437

**Published:** 2024-02-13

**Authors:** Plinio P. Morita, Matheus Lotto, Jasleen Kaur, Dmytro Chumachenko, Arlene Oetomo, Kristopher Dylan Espiritu, Irfhana Zakir Hussain

**Affiliations:** ^1^School of Public Health Sciences, University of Waterloo, Waterloo, ON, Canada; ^2^Department of Systems Design Engineering, University of Waterloo, Waterloo, ON, Canada; ^3^Research Institute for Aging, University of Waterloo, Waterloo, ON, Canada; ^4^Centre for Digital Therapeutics, Techna Institute, University Health Network, Toronto, ON, Canada; ^5^Institute of Health Policy, Management, and Evaluation, Dalla Lana School of Public Health, University of Toronto, Toronto, ON, Canada; ^6^Department of Pediatric Dentistry, Orthodontics, and Public Health, Bauru School of Dentistry, University of São Paulo, Bauru, Brazil; ^7^Department of Mathematical Modelling and Artificial Intelligence, National Aerospace University “Kharkiv Aviation Institute”, Kharkiv, Ukraine

**Keywords:** artificial intelligence, infodemic, health information management, misinformation, eHealth

## Abstract

Artificial intelligence (AI) chatbots have the potential to revolutionize online health information-seeking behavior by delivering up-to-date information on a wide range of health topics. They generate personalized responses to user queries through their ability to process extensive amounts of text, analyze trends, and generate natural language responses. Chatbots can manage infodemic by debunking online health misinformation on a large scale. Nevertheless, system accuracy remains technically challenging. Chatbots require training on diverse and representative datasets, security to protect against malicious actors, and updates to keep up-to-date on scientific progress. Therefore, although AI chatbots hold significant potential in assisting infodemic management, it is essential to approach their outputs with caution due to their current limitations.

## Introduction

The widespread availability of Internet access has led to its use as a primary source of health information. Online health information-seeking behavior goes beyond searching for symptoms, diagnoses, and treatments for specific conditions (1, 2); self-care and healthy lifestyle choices have become increasingly prioritized. Digital resources empower individuals to make informed health decisions and contribute to a more equitable and accessible healthcare landscape (3). Additionally, the vast reservoir of big data stemming from individuals’ online health-seeking behavior also possesses the potential to provide insights for public health decisions (4). Researchers access this big data through public APIs, collaborations with platform providers, or web scraping tools, ensuring that user privacy and anonymity are maintained. Such data becomes foundational in Infodemiology, revealing patterns in health information dissemination and consumption. Infodemiology comes to the fore as a scientific framework with a core focus on analyzing the dissemination and influence of information within electronic platforms, particularly on the Internet, and across diverse populations (5). As a result, its ultimate goal is to enhance public health awareness and guide the trajectory of public policy through well-founded insights (5).

On the other hand, contemporary information ecosystems are rife with content overabundance. This presents a challenge to the average user who must navigate and screen for relevant information. The situation is further complicated by the fact that online health-related content is often incomplete, outdated, misleading, or false (6, 7). Consequently, people are increasingly vulnerable to the consumption of health misinformation (8). Perpetuation of negative health beliefs can effectively reverse any benefits arising from digital information-seeking behavior (7, 9).

Major health events, such as the COVID-19 pandemic, are often accompanied by an infodemic: an overwhelming amount of information and misinformation that spreads rapidly through digital and physical environments (10, 11). Misinformation arising during infodemic can have serious negative consequences for public health. For instance, COVID-19-related misinformation delayed vaccination rates among American and British individuals (12). There is an increasing need to manage digital health information to mitigate loss due to misinformation.

Infodemic management involves systematically using risk-and evidence-based analysis and approaches to soften the impacts of misinformation on people’s health behavior (9). While mitigating misinformation gained particular notoriety during the COVID-19 pandemic, it remains crucial in health fields plagued by falsehoods: vaccination, fluoridation, drugs, and smoking, noncommunicable diseases, eating disorders, and medical treatments (7, 13). Specifically, researchers can capitalize on the extensive pool of big data from individuals seeking health information to support infodemic management. This encompasses exploring data sources such as search engine tools, social media platforms, and interactions with chatbots.

## Chatbots as health information mediators

Individuals have previously relied on search engine tools (Google, Bing, Yahoo!) and social media platforms (Facebook, Instagram, Twitter) to address their queries, concerns, and doubts concerning health-related topics (7, 14, 15). The development of new artificial intelligence (AI)-based interactive chatbots (i.g. OpenAI’s ChatGPT) offers several advantages over traditional health information search platforms. People use AI chatbots to search for health information by typing their queries into the chatbot interface. Chatbots then analyze and summarize large volumes of text from a variety of sources and generate natural language responses. Users can also provide additional information about their location, age, and other relevant factors to personalize the response. This makes it an ideal tool for engaging with the public and responding to questions and concerns in real-time, providing users with personalized, meaningful conversations on various health topics (16). Theoretically, chatbots also possess the capability to debunk common myths and misconceptions, promoting accurate information (17, 18). As a result, they could provide more precise and up-to-date information on health-related topics (17, 18). An additional advantage over traditional search engines is their ability to interact with public health actions and be integrated with other digital health tools. As such, chatbots could represent a valuable public health tool in infodemic management and public health (19).

## Technology challenges

**Figure 1:**
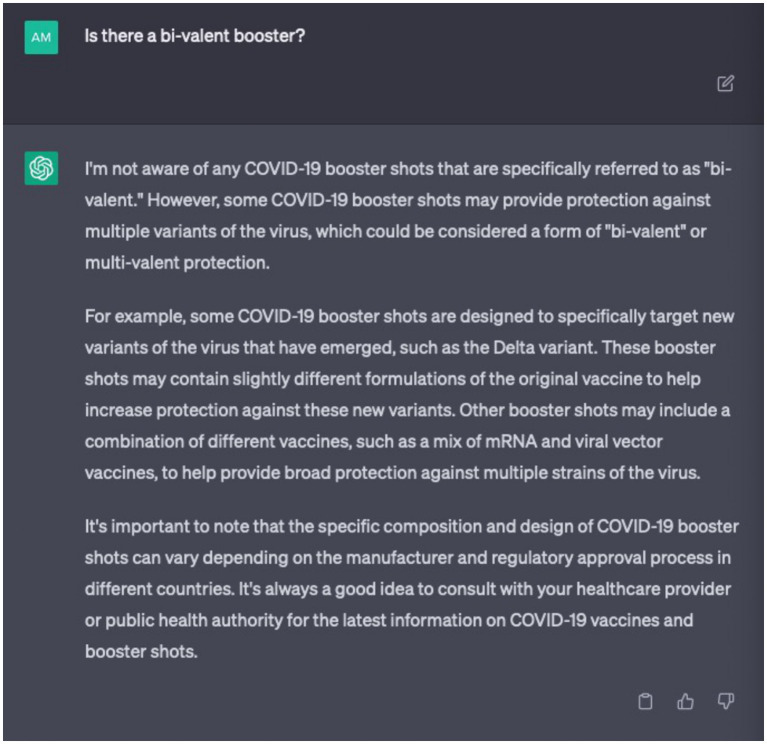
ChatGPT-generated response exemplifying a potential occurrence of outdated information.

Chatbot development must overcome several technical challenges before their widespread implementation for infodemic management. As AI chatbots rely on machine learning algorithms, their accuracy can be affected by the quality of the training data and the complexity of the queries they receive. Large language models like chatbots fail in complex open-domain questions due to comprehension errors, factualness, specificity, and inference (20). One of the main challenges is ensuring that the model is trained on a diverse and representative dataset to avoid bias and confirm that it can accurately understand and generate language across a range of contexts and demographics. AI chatbots can sometimes generate false or misleading information, especially if they are not properly monitored and maintained (21). In this domain, certain instances manifest wherein these chatbots either abstain from furnishing corroborative references for their discourse or produce references that are erroneous in their information attribution. Undoubtedly, relying on outdated content for training purposes inevitably leads to generating obsolete outputs. This is a significant challenge when scientific evidence evolves rapidly as seen during the COVID-19 pandemic. For instance, if ChatGPT 3 had a knowledge cut-off in September 2021, a query of “Is there a bi-valent booster?” placed in April 2023 would return the erroneous result that it is not aware of any COVID-19 booster shots (Figure 1). In this case, outdated training content could potentially cause harm and create confusion. As such, chatbots could not only fail to assist in infodemic management but may worsen it. Another challenge is ensuring that the model is secure and protected from malicious actors who might seek to manipulate it for their own purposes.

To tackle these challenges, developers must consistently train AI models with high-quality data and implement error detection and correction measures (22). This process encompasses: (i) gathering new data, preprocessing, and retraining; (ii) transfer learning to incorporate new knowledge while retaining past understanding in training new chatbot versions; (iii) updating dialog management to accommodate novel intents and contexts; (iv) conducting regular testing and validation to adapt responses accordingly; (v) establishing a feedback loop to drive improvements based on user perspectives; (vi) maintaining continuous monitoring to address post-update issues promptly; (vii) scheduling periodic updates to align training with evolving information and user requirements; and (viii) ensuring human oversight to scrutinize responses after significant updates for precision. In practical terms, if they have been trained with reliable information grounded in high-level scientific evidence, they are unlikely to propagate misinformation on the subject at hand. Finally, model transparency is imperative, as all algorithms are susceptible to bias.

Chatbots represent sophisticated tools that demand vigilant supervision by experts in both health and technology domains. Significantly, health professionals overseeing these systems should be abreast of the latest advancements in scientific evidence to mitigate the risk of inadvertently endorsing inaccuracies. There is a valid apprehension that, in the rush to introduce digital products, inadequately developed tools with significant deficiencies upon release may contribute to adverse consequences. The recent proliferation of such tools poses a potential disaster in the ongoing battle against misinformation, underscoring the critical need for enhanced oversight measures.

## Regulation of AI chatbots

While generative AI chatbots exhibit potential in debunking misinformation, it is imperative to acknowledge that they may disseminate falsehoods or outdated concepts if not regularly updated with a foundation based on a high level of scientific evidence. Additionally, there exists the prospect of conflicts of interest favoring the propagation of misinformation on these platforms, as previously observed in social media, driven by hidden financial or political motivations (7). Consequently, the imperative lies in enforcing stringent regulations governing the operation of these chatbots, ensuring the safeguarding of the interests of public health and the population. Essential elements within policies and guidelines should encompass delineating the responsibilities and liabilities of chatbot platforms, upholding ethical principles, ensuring transparency, fostering active collaboration with stakeholders, prioritizing data privacy and security, and demonstrating an unwavering commitment to effectively curbing health falsehoods. In this regard, the European Union is in the process of finalizing legislation on AI that promises to tightly regulate chatbots. The EU’s AI Act mandates the adherence of these systems to a delineated set of transparency principles, encompassing compliance with European Union copyright law and the formulation of comprehensive summaries delineating the content employed in training AI models, as articulated in the official statement from the European Parliament. High-impact models deemed to pose systemic risks will face stricter regulations, including the requirement to assess and mitigate such risks. Violations of these regulations may incur penalties of up to 7% of a company’s global revenue, contingent upon the size of the entity and the specific contravened rule, under the provisions outlined in the official declaration. Evidently, this foundational stride ought to be extrapolated to encompass diverse nations, preserving their idiosyncrasies while upholding their fundamental objectives.

## Concluding remarks

The emergence of chatbot technology has the potential to revolutionize users’ online health information-seeking behavior; positively impacting the infodemic and, ultimately, public health. Generative AI chatbots can reduce misinformation by providing credible and reliable sources of information. Additionally, they can be trained to recognize falsehoods, fact-check information, and provide personalized responses to user queries. Nevertheless, they require expertise in their management and high-quality training sets to ensure their efficacy, and it is essential to approach their outputs with caution due to their inherent limitations. Consequently, there exists a critical need to institute comprehensive regulation governing their usage, underpinned by the importance of regular updates. These measures are crucial in cultivating a secure ecosystem for health information access (23). Future studies should prioritize managing misinformation within chatbot platforms while emphasizing the importance of promoting trustworthiness in health information-seeking behavior.

## Data availability statement

The original contributions presented in the study are included in the article/supplementary material, further inquiries can be directed to the corresponding author.

## Author contributions

PM: Conceptualization, Writing – review & editing. ML: Writing – original draft. JK: Writing – original draft. DC: Writing – original draft. AO: Writing – original draft. KE: Writing – original draft. IH: Writing – original draft.
